# Case Report: Autoimmune hepatitis following oxaliplatin exposure presenting with multifactorial non-cirrhotic portal hypertension and recurrent parastomal variceal bleeding

**DOI:** 10.3389/fmed.2026.1857471

**Published:** 2026-09-04

**Authors:** Wei Cheng, Hongbo Shi, Jiaju Fan, Xiaoming He, Haibin Shi, Yi Li, Yunfeng Huan, Shengqin Zhang

**Affiliations:** 1 Department of Gastrointestinal Surgery, The First Affiliated Hospital of Dali University, Dali, Yunnan, China; 2 Department of Gynaecology, The First Affiliated Hospital of Dali University, Dali, Yunnan, China; 3 Department of Vascular Surgery, The First Affiliated Hospital of Dali University, Dali, Yunnan, China

**Keywords:** autoimmune hepatitis, non-cirrhotic portal hypertension, oxaliplatin, parastomal varices, portal hypertension

## Abstract

Ectopic variceal hemorrhage accounts for approximately 5% of all variceal bleeding episodes and represents an uncommon manifestation of portal hypertension. This report describes recurrent parastomal variceal bleeding in a 43-year-old woman with rectal adenocarcinoma who underwent abdominoperineal resection followed by five cycles of oxaliplatin-containing chemotherapy. During chemotherapy, severe hepatocellular injury developed. A liver biopsy performed in June 2020 demonstrated severe interface hepatitis with lymphoplasmacytic portal inflammation, findings that supported a diagnosis of autoimmune hepatitis (AIH). No histopathological features diagnostic of portosinusoidal vascular disease (PSVD) were identified. Subsequently, progressive splenomegaly, gastroesophageal and parastomal varices, and a directly measured portal pressure of 35 cm H_2_O developed in the absence of established cirrhosis. Inferior mesenteric vein embolization and ultrasound-guided foam sclerotherapy reduced the frequency of recurrent parastomal variceal bleeding but did not achieve complete hemostatic control. Treatment with carvedilol and prednisone was subsequently initiated. The overall clinical presentation was most consistent with multifactorial non-cirrhotic portal hypertension associated with AIH and prior oxaliplatin exposure, with a potential contribution from oxaliplatin-associated sinusoidal or vascular liver injury rather than histologically confirmed PSVD. This case highlights the diagnostic complexity and the need for individualized management in individuals with coexisting autoimmune liver disease, chemotherapy-associated hepatic injury, and refractory ectopic variceal hemorrhage.

## 1 Introduction

Ectopic variceal hemorrhage accounts for approximately 5% of all variceal bleeding episodes and occurs at sites outside the gastroesophageal region (1). Among individuals with colorectal cancer who undergo ostomy creation, parastomal varices may develop as a result of collateral venous circulation within the abdominal wall (2). Oxaliplatin, a platinum-based chemotherapeutic agent widely used in the treatment of colorectal cancer, is associated with sinusoidal liver injury, including sinusoidal obstruction syndrome/veno-occlusive disease (SOS/VOD), and may contribute to the development of portal hypertension (3–5). Portosinusoidal vascular disease (PSVD) encompasses a spectrum of hepatic vascular disorders that occur in the absence of established cirrhosis. Characteristic histopathological features include obliterative portal venopathy, nodular regenerative hyperplasia, and incomplete septal or perisinusoidal fibrosis (6, 7). However, portal hypertension occurring after oxaliplatin exposure should not be considered synonymous with PSVD in the absence of diagnostic histopathological findings. This report describes a patient with biopsy-supported autoimmune hepatitis (AIH) following oxaliplatin exposure who subsequently developed multifactorial non-cirrhotic portal hypertension and recurrent parastomal variceal hemorrhage.

## 2 Case data

### 2.1 Disease history

A 43-year-old woman with a history of hypothyroidism, intermittently treated with levothyroxine sodium (25 μg once daily), presented without a history of viral hepatitis, relevant family history, or known drug or food allergies. Colonoscopy performed in June 2019 identified rectoanal carcinoma. On July 6, 2019, the patient underwent laparoscopic abdominoperineal resection with permanent colostomy (Miles procedure). Postoperative histopathological examination confirmed moderately differentiated ulcerative tubular adenocarcinoma of the rectum, staged as pT3N1bM0, corresponding to American Joint Committee on Cancer (AJCC) stage IIIB.

Adjuvant chemotherapy was initiated with capecitabine plus oxaliplatin (oxaliplatin 130 mg/m^2^ administered intravenously on day 1 and capecitabine 1,000 mg/m^2^ administered orally on days 1–14 of a 3-week cycle). Capecitabine was discontinued after the first cycle because of palmar-plantar erythrodysesthesia. Oxaliplatin monotherapy was administered on August 21, 2019, after which the regimen was changed to modified FOLFOX6 (mFOLFOX6) on September 16, 2019. Available medical records documented five cycles of oxaliplatin-containing chemotherapy, corresponding to an estimated cumulative oxaliplatin dose of approximately 750 mg/m^2^.

Marked elevations in liver enzyme levels were first documented during the third chemotherapy cycle [September 16, 2019: alanine aminotransferase (ALT), 910 U/L; aspartate aminotransferase (AST), 1,151 U/L]. A fourth cycle of mFOLFOX6 was administered on October 9, 2019. Although liver enzyme levels subsequently declined, they remained above the reference range. A final oxaliplatin-containing treatment was administered on November 4, 2019, followed by another marked increase in liver enzyme levels (November 7, 2019: ALT, 839 U/L; AST, 703 U/L). The patient was hospitalized on multiple occasions for evaluation and management of suspected drug-induced liver injury. The chronological sequence of events is presented in Table 1, and detailed laboratory findings are presented in Supplementary Table S1.

**Table 1:** Chronological timeline of chemotherapy, liver injury, portal hypertension, and therapeutic interventions.

Date/period	Clinical event and key findings
June–July 2019	Rectal adenocarcinoma diagnosed. Laparoscopic abdominoperineal resection (Miles procedure) with permanent colostomy performed on July 6, 2019.
August–November 2019	Five cycles of oxaliplatin-containing chemotherapy administered (estimated cumulative oxaliplatin dose approximately 750 mg/m^2^). Severe hepatocellular injury developed during the third cycle and recurred following the final documented oxaliplatin exposure.
Approximately February 2020	First documented episode of parastomal variceal hemorrhage, approximately seven months after surgery.
June 2020	Percutaneous liver biopsy demonstrated severe interface hepatitis with prominent lymphoplasmacytic portal inflammation, supporting a diagnosis of AIH. No diagnostic histopathological features of PSVD lesions were identified.
2020–2022	Progressive splenomegaly with development of gastroesophageal and parastomal varices.
June 28, 2022	Portal venography, direct portal venous pressure measurement (35 cm H₂O), inferior mesenteric vein sclerotherapy, and coil embolization performed.
September 9, 2022	Ultrasound-guided percutaneous foam sclerotherapy of peristomal varices performed.
June 2023 onward	Markedly elevated serum IgG concentrations and positive SLA/ANA test results documented. Treatment with carvedilol and prednisone initiated.
January 2025	Gastroesophageal variceal hemorrhage treated with emergency endoscopic variceal ligation. TIPS placement was reconsidered but declined.
Latest follow-up	No recurrent upper gastrointestinal hemorrhage documented. Parastomal variceal hemorrhage decreased to approximately one episode every two months.

A percutaneous liver biopsy performed in June 2020 demonstrated marked lymphoplasmacytic infiltration of the portal tracts, small bile duct injury with ductular reaction, severe interface hepatitis, intralobular lymphocytic infiltration, and spotty hepatocellular necrosis. These findings supported a diagnosis of AIH but were not sufficiently specific to exclude drug-induced autoimmune-like hepatitis. No histopathological evidence of obliterative portal venopathy, nodular regenerative hyperplasia, or marked perisinusoidal fibrosis diagnostic of PSVD was identified. Representative histopathological findings are presented in Supplementary Figure S1. Special histochemical staining yielded the following results: silver (+), Masson (+), PAS (+), D-PAS (−), VG (+), hemosiderin (−), and rhodanine (−). Immunohistochemical staining was positive for CD4, CD8, CD20, Mum-1, IgG, IgM, CK7 (cholangiocytes), and CK19 (cholangiocytes). Initial treatment with ursodeoxycholic acid and compound glycyrrhizinate did not achieve complete biochemical remission.

**Figure 1:**
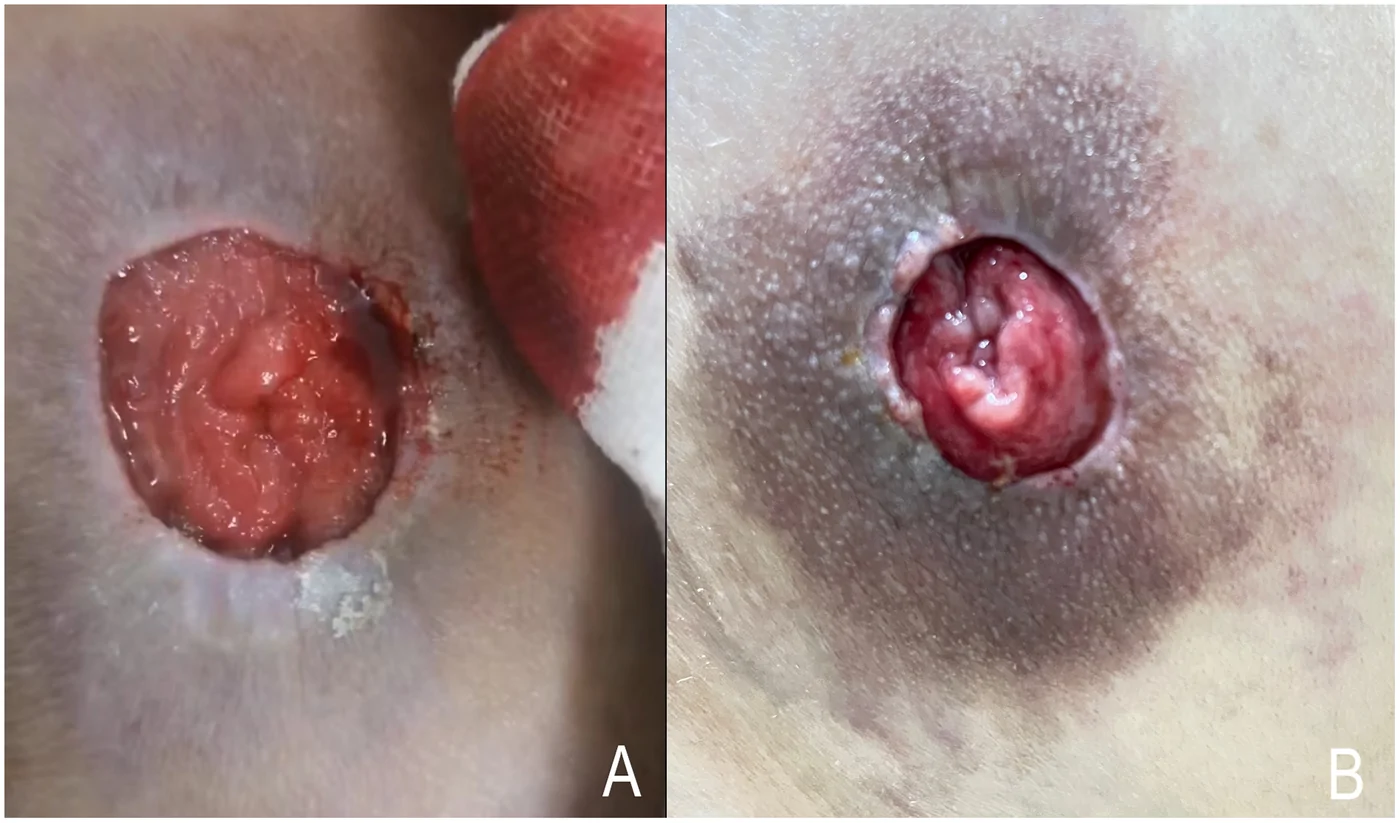
Parastomal variceal hemorrhage and abdominal wall varices. **(A)** Hemorrhage originating from the mucocutaneous junction of the colostomy. **(B)** Peristomal hyperpigmentation corresponding to the distribution of abdominal wall varices.

Approximately 7 months after surgery, parastomal hemorrhage was observed at the colostomy site (Figure 1A). Approximately 2 years after surgery, abdominal wall varices developed around the stoma (Figure 1B). On June 28, 2022, the patient underwent portal venous puncture angiography, ultra-selective inferior mesenteric vein sclerotherapy with sclerosant injection, coil embolization, portal vein pressure measurement, and coronary vein coil embolization. Despite these interventions, recurrent parastomal hemorrhage recurred 20 days later and progressively worsened until September 2022, when ultrasound-guided percutaneous foam sclerotherapy of the abdominal wall varices was performed. Intermittent parastomal hemorrhage persisted following the procedure, although no severe or life-threatening bleeding episodes were documented.

On June 13, 2023, the patient was admitted to the Department of Rheumatology and Immunology for evaluation of an 8-month history of recurrent bilateral lower-extremity rash. Since November 2022, spontaneous scattered petechiae had developed on both lower extremities. The lesions were initially sparse and resolved spontaneously without specific treatment. However, the rash progressively worsened in May 2023, resulting in substantial impairment of ambulation. The patient was diagnosed with allergic purpura in the dermatology outpatient clinic and treated with oral dipyridamole, vitamins C and E, compound glycyrrhizinate, and self-administered loratadine. Partial improvement was achieved, although recurrent episodes persisted.

Additional immunological investigations were performed during this hospitalization. The tear break-up time (BUT) was within the normal range (> 14 s). A labial salivary gland biopsy demonstrated a small amount of glandular tissue with scattered interstitial lymphocytic infiltration and no evidence of acinar atrophy, findings that did not satisfy the diagnostic criteria for Sjögren's syndrome. Furthermore, the patient did not report characteristic sicca symptoms, including xerostomia, xerophthalmia, or an increased incidence of dental caries. Collectively, the available clinical and pathological findings did not support a diagnosis of a definitive systemic immune-mediated disease.

Laboratory investigations performed in June 2023 demonstrated an IgG concentration of 52.70 g/L, anti-soluble liver antigen (SLA) antibody > 400.00 RU/mL, antinuclear antibody (ANA) > 400.00 RU/mL, anti-Ro-52 antibody > 400 RU/mL, and reduced complement C4 (0.054 g/L). Antimitochondrial antibody M2 (AMA-M2) values of 23.07 and 25.73 RU/mL were documented in the medical record, with a subsequent value of 40.42 RU/mL. Because assay-specific cutoff values and contemporaneous confirmatory testing were unavailable, these results were not interpreted as definitively positive or negative. Treatment with compound glycyrrhizin, ursodeoxycholic acid, polyene phosphatidylcholine, total glucosides of paeony, and cetirizine resulted in improvement of the skin rash. However, no definitive systemic rheumatic disease was diagnosed.

### 2.2 Auxiliary examination

During multiple hospitalizations, physical examination demonstrated no jaundice of the skin or sclerae, palmar erythema, or spider angiomas. Cardiopulmonary examination was unremarkable. Approximately two years after surgery, hyperpigmentation developed around the colostomy site, corresponding to the presence of abdominal wall varices (Figure 1B). Parastomal hemorrhage most frequently originated at the mucocutaneous junction of the stoma (Figure 1).

Abdominal examination revealed no tenderness, rebound tenderness, or guarding. The liver and spleen were not palpable below the costal margins, and no clinical evidence of ascites, including shifting dullness, was detected. No peripheral edema was present in either lower extremity.

**Figure 2:**
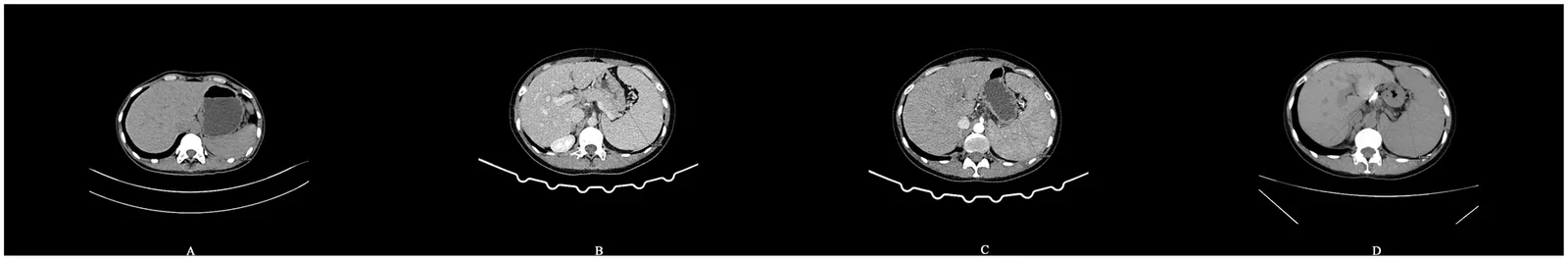
Serial non-contrast and contrast-enhanced CT examinations demonstrating progressive splenomegaly. **(A)**: July 2019; **(B)**: June 2022; **(C)**: June 2023; **(D)**: January 2025. Yellow text indicates the approximate spleen measurement(cm).

**Figure 3:**
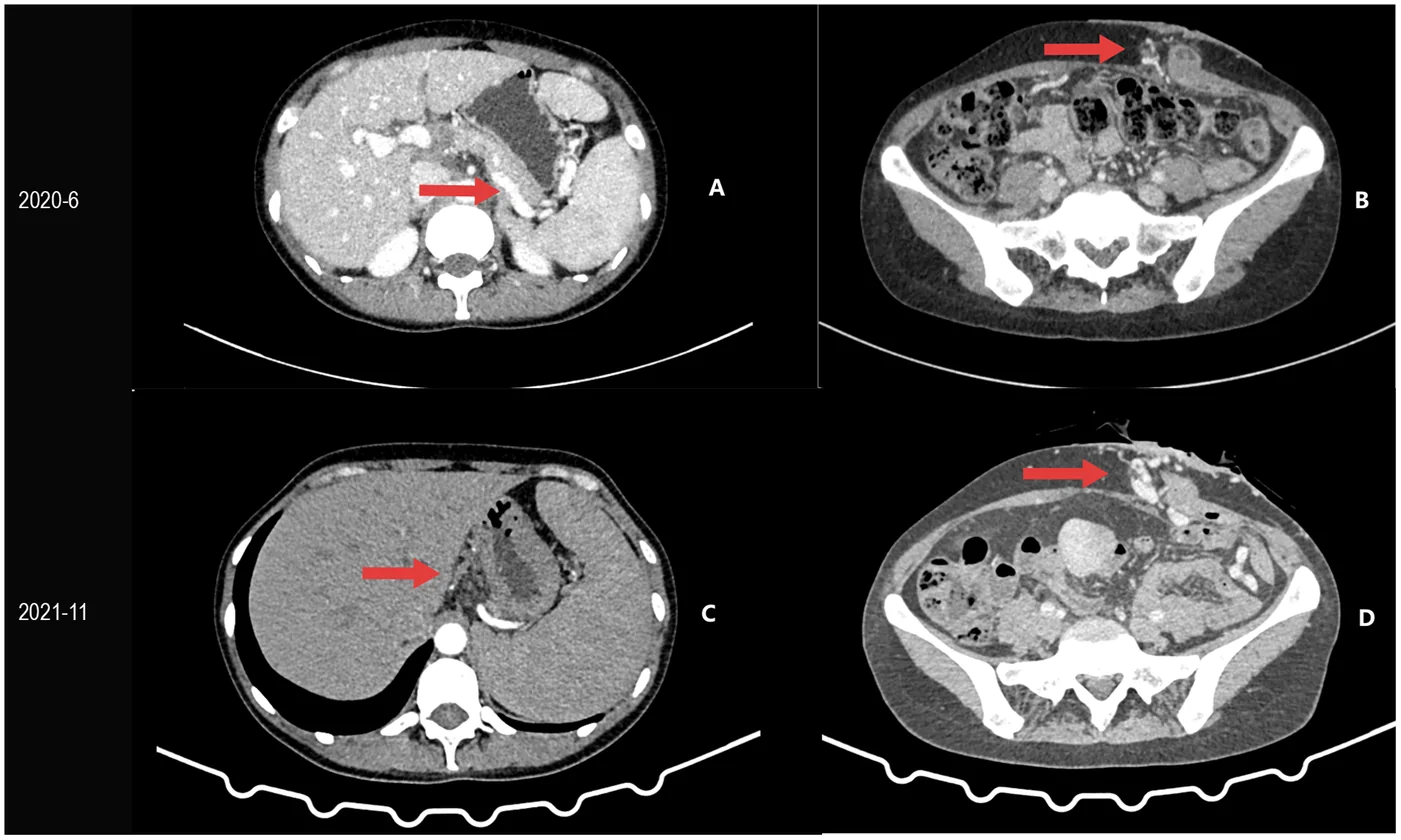
Contrast-enhanced CT demonstrating features of portal hypertension, including gastroesophageal varices and tortuous peristomal collateral vessels. **(A)** The red arrow indicates tortuous and dilated splenic vessels; **(B)** The red arrow indicates tortuous peristomal collateral veins; **(C)** The red arrow indicates dilated gastroesophageal varices adjacent to the gastric fundus; **(D)** The red arrow indicates progression of the peristomal collateral veins compared with the previous examination.

Serial liver function test results are presented in Table 2. Recurrent episodes of hepatocellular injury were observed, whereas serum bilirubin, albumin, and the international normalized ratio (INR) remained generally preserved. Platelet counts gradually declined from within the reference range to 121 × 10^9^/L by March 2025. Progressive splenomegaly was documented on serial non-contrast and contrast-enhanced computed tomography (CT) examinations performed before and after chemotherapy (Figure 2). Contrast-enhanced CT demonstrated features of portal hypertension, including gastroesophageal varices and tortuous dilated peristomal collateral vessels (Figure 3). Key longitudinal laboratory findings are presented in Supplementary Table S1.

**Table 2:** Serial liver biochemical test results during clinical follow-up.

Parameter (U/L)	2019-09-17	2019-10-10	2019-10-18	2019-11-5	2019-11-29	2020-03-04	2020-06-02	2021-01-19	2021-11-2
ALT/	-	716	189	637	839	279	144	66	80
AST/	1151	706	121	528	703	236	445	63	69
GGT/	-	316	-	176	184	116	182	18	22

ALT, alanine aminotransferase; AST, aspartate aminotransferase; GGT, gamma-glutamyl transferase.

Ultrasonography demonstrated markedly dilated, tortuous peristomal veins with a honeycomb-like appearance. The largest vessel measured approximately 3 mm in diameter.

### 2.3 Treatment and outcome

On June 28, 2022, the patient underwent portal venous puncture angiography, ultra-selective inferior mesenteric vein sclerotherapy, coil embolization, portal vein pressure measurement, and coronary vein coil embolization. Under B-mode ultrasonographic guidance, the portal vein was punctured, and contrast medium was administered through a 5-F vascular sheath. Digital subtraction angiography was subsequently performed for portal venography. Imaging demonstrated dilatation of the portal vein and its branches, gastric coronary vein varices, and a portal venous pressure of 35 cm H₂O (approximately 25.7 mmHg), confirming clinically significant portal hypertension. This value represented an absolute portal venous pressure obtained by direct percutaneous transhepatic measurement, rather than a hepatic venous pressure gradient (HVPG). Free hepatic venous pressure and wedged hepatic venous pressure were not measured; therefore, HVPG was not calculated in this patient.

A C2 catheter and loach guidewire were advanced ultra-selectively into the inferior mesenteric vein. Angiography demonstrated tortuous, dilated veins surrounding the colostomy site arising from the distal inferior mesenteric vein, with clustered varices (Supplementary Figure S2). A 5-F angled catheter and microcatheter were then advanced into the variceal vessels. Foam sclerosant was injected through the microcatheter, followed by deployment of two detachable coils to embolize the distal inferior mesenteric vein. Post-embolization angiography demonstrated reduced opacification of the distal inferior mesenteric vein.

On September 9, 2022, ultrasound-guided percutaneous foam sclerotherapy of the abdominal wall varices was performed. Ultrasonography demonstrated clustered, tortuous, and dilated peristomal veins, with the largest measuring approximately 3 mm in diameter. Foam sclerosant was prepared by mixing 2 mL of polidocanol with air at a 1:5 ratio using two 10-mL syringes to produce a homogeneous foam. More than 10 variceal sites were punctured under ultrasonographic guidance, and approximately 0.5 mL of foam sclerosant was injected into each site. Post-procedural ultrasonography demonstrated homogeneous distribution of the sclerosant, marked reduction in blood flow velocity, and absence of detectable blood flow within several treated vessels.

Carvedilol was initiated at a dose of 6.25 mg once daily to reduce portal pressure. Prednisone was initiated at 40 mg/day, tapered by 5 mg weekly after the initial 2 weeks, and maintained at 12.5 mg/day. Thiopurine methyltransferase (TPMT) testing was not performed. Although azathioprine and mycophenolate mofetil were considered as steroid-sparing immunosuppressive agents, neither was initiated because of the patient's concerns regarding potential adverse effects and preference for corticosteroid monotherapy. Available follow-up data demonstrated preserved hepatic synthetic function. However, serial IgG measurements and a complete contemporaneous biochemical dataset following prednisone initiation were unavailable in the reviewed medical records. Consequently, complete biochemical remission and the adequacy of long-term immunosuppressive therapy could not be confirmed.

During follow-up, serum bilirubin, albumin, and INR remained generally preserved, whereas mild intermittent elevations in aminotransferase levels and thrombocytopenia persisted. Because serial IgG measurements were unavailable, the biochemical response of AIH to immunosuppressive therapy could not be comprehensively evaluated.

In January 2025, the patient was hospitalized for upper gastrointestinal bleeding. CT demonstrated esophageal and gastric varices with a small volume of pelvic free fluid, and emergency upper gastrointestinal endoscopy confirmed bleeding gastroesophageal varices. Endoscopic variceal ligation successfully achieved hemostasis. Following this event, transjugular intrahepatic portosystemic shunt (TIPS) placement was reconsidered; however, the patient declined the procedure because of concerns regarding procedural risks and uncertain clinical benefit. No recurrent upper gastrointestinal bleeding had been documented at the most recent follow-up.

Before inferior mesenteric vein embolization and percutaneous foam sclerotherapy, parastomal hemorrhage occurred one to two times daily. Following treatment, bleeding ceased for approximately 18 months and subsequently recurred intermittently at a substantially lower frequency of approximately once every 2 months. Longitudinal changes in hemoglobin concentration, red blood cell count, and platelet count are presented in Supplementary Figure S3.

## 3 Discussion

### 3.1 Etiology and differential diagnosis of liver injury and portal hypertension

In the present case, liver injury developed during oxaliplatin-based chemotherapy. During subsequent follow-up, progressive splenomegaly, gastroesophageal varices, and recurrent parastomal variceal bleeding developed, confirming the presence of clinically significant portal hypertension. The constellation of clinical, laboratory, imaging, and histopathological findings was compatible with several potential etiologies, making the differential diagnosis challenging. Established cirrhosis was not supported by the available imaging, histopathological, or laboratory findings. Accordingly, prehepatic, posthepatic, and intrahepatic causes of non-cirrhotic portal hypertension were systematically considered.

Prehepatic and posthepatic causes were evaluated using the available clinical and imaging data. Contrast-enhanced abdominal CT demonstrated no thrombosis or major obstructive lesions involving the main portal vein, splenic vein, or superior mesenteric vein. Likewise, no evidence of hepatic venous outflow obstruction or inferior vena cava obstruction was identified. The patient had no history of right-sided heart failure or constrictive pericarditis and exhibited no persistent ascites, lower-extremity edema, or other manifestations of systemic venous congestion. Collectively, these findings made chronic extrahepatic portal vein obstruction and major posthepatic causes unlikely. However, comprehensive vascular and thrombophilia evaluation were not performed and therefore cannot be excluded definitively.

Following exclusion of these conditions, the principal intrahepatic etiologies were evaluated on the basis of the available clinical, imaging, serological, and histopathological evidence, as discussed below.

1. Oxaliplatin exposure: Oxaliplatin is a well-recognized cause of sinusoidal liver injury and has been associated with sinusoidal obstruction syndrome (SOS), chronic vascular liver injury, and non-cirrhotic portal hypertension, including cases classified as PSVD or idiopathic non-cirrhotic portal hypertension (3–5, 8). In the present case, the temporal association relationship between oxaliplatin exposure and liver injury was noteworthy. Available medical records documented five cycles of oxaliplatin-containing chemotherapy and an estimated cumulative oxaliplatin dose of approximately 750 mg/m2, although complete records of dose modifications for each cycle were unavailable. Marked hepatocellular injury developed during the third chemotherapy cycle (ALT, 910 U/L; AST, 1,151 U/L), improved after the fourth cycle, and recurred following the final documented oxaliplatin exposure (ALT, 839 U/L; AST, 703 U/L). The risk of oxaliplatin-associated sinusoidal injury has been demonstrated to increase with cumulative exposure, and previous studies have demonstrated dose-dependent increases in splenic volume during treatment (4, 5, 9–14). However, these population-level associations do not establish causality in an individual patient.
2. AIH: The patient demonstrated strongly positive anti-soluble liver antigen (SLA) antibodies (> 400 RU/mL), ANA > 400 RU/mL, and markedly elevated serum IgG (52.70 g/L). Liver biopsy demonstrated severe interface hepatitis with prominent lymphoplasmacytic portal inflammation. According to the simplified International Autoimmune Hepatitis Group (IAIHG) criteria, the available findings yielded a score of at least 7 points: anti-SLA positivity (2 points), serum IgG > 1.1 times the upper limit of normal (2 points), histology compatible with AIH (1 point), and exclusion of viral hepatitis (2 points). This score fulfills the diagnostic threshold for definite AIH (15, 16). The detailed scoring system is presented in Supplementary Table S2. Nevertheless, anti-SLA positivity and markedly elevated serum IgG concentrations were documented in 2023 rather than at the onset of chemotherapy-associated liver injury. Accordingly, although the available evidence supports a diagnosis of AIH, it does not establish AIH as the sole cause of the initial hepatic injury.
3. PSVD: The clinical manifestations, including progressive splenomegaly, variceal hemorrhage, and elevated portal pressure, were consistent with non-cirrhotic portal hypertension and raised the possibility of PSVD. However, the available liver biopsy did not demonstrate characteristic histopathological features of PSVD, including obliterative portal venopathy, nodular regenerative hyperplasia, or marked perisinusoidal fibrosis. Although sampling error cannot be excluded and the patient declined repeat liver biopsy, histopathological confirmation of PSVD was not established. Furthermore, distinguishing portal hypertension associated with AIH and PSVD may be challenging because of overlapping pathological features (17). Therefore, the available evidence is most consistent with multifactorial non-cirrhotic portal hypertension in associated with AIH and prior oxaliplatin exposure, with a possible contribution from oxaliplatin-associated sinusoidal or vascular injury rather than histologically confirmed PSVD.
4. AIH-primary biliary cholangitis (PBC) overlap syndrome: AMA-M2 values of 23.07, 25.73, and 40.42 RU/mL were documented. However, assay-specific reference ranges and contemporaneous confirmatory testing were unavailable; consequently, these results could not be interpreted reliably as either positive or negative. Although liver biopsy demonstrated small bile duct injury with ductular reaction, neither florid duct lesions nor ductopenia was identified, and a persistent cholestatic biochemical profile was not documented. Therefore, the available evidence was insufficient to establish a diagnosis of AIH-PBC overlap syndrome, although this possibility could not be completely excluded.
5. Drug-induced autoimmune-like hepatitis (DI-ALH): Drug-induced autoimmune-like hepatitis may closely resemble idiopathic AIH with respect to its clinical, serological, and histopathological features. In the present case, liver injury developed during oxaliplatin exposure, whereas anti-SLA positivity and markedly elevated serum IgG concentrations were documented subsequently. Although severe interface hepatitis and lymphoplasmacytic portal inflammation support a diagnosis of AIH, these findings do not exclude DI-ALH. Consequently, the available evidence does not permit definitive differentiation among pre-existing AIH, oxaliplatin-triggered or oxaliplatin-exacerbated AIH, and DI-ALH.
6. Nodular regenerative hyperplasia (NRH): NRH represents a recognized histopathological pattern within the PSVD spectrum and may occur in association with drug exposure or immune-mediated disorders. However, the available liver biopsy did not demonstrate regenerative nodules characteristic of NRH, and imaging studies did not reveal features indicative of this condition. Accordingly, NRH was not established.
7. Progression of inadequately treated AIH to cirrhosis: Progression to cirrhosis remained a clinically relevant diagnostic consideration. Serial imaging demonstrated a smooth hepatic contour without established nodularity, while serum albumin concentrations and INR remained generally preserved. These findings argue against established cirrhosis but do not exclude progressive hepatic fibrosis, particularly because comprehensive fibrosis assessment, including transient elastography, hepatic venous pressure gradient (HVPG) measurement, and repeat liver biopsy, was unavailable. Importantly, portal hypertension-associated splenomegaly and varices should not be interpreted in isolation as evidence of cirrhosis.

Taken together, the available evidence supports a multifactorial etiology for both the liver injury and portal hypertension. AIH was likely the principal contributor to chronic hepatocellular inflammation, whereas oxaliplatin-associated sinusoidal or vascular injury may have contributed either jointly or independently to increased intrahepatic vascular resistance. This multifactorial process provides a plausible explanation for the progressive splenomegaly, development of gastroesophageal and parastomal varices, and refractory parastomal variceal hemorrhage observed in this patient. However, the available evidence does not establish a single causal mechanism or histopathologically confirmed PSVD.

Several limitations should be acknowledged. Repeat liver biopsy, transient elastography, and HVPG measurement were not performed because the patient declined these investigations. In addition, a comprehensive thrombophilia evaluation, including testing for the *JAK2* V617F variant and antiphospholipid antibodies, was not undertaken. Although no portal or splanchnic venous thrombosis was identified on the available imaging studies, the absence of a complete thrombophilia work-up limits the etiological assessment.

### 3.2 Treatment challenges and management strategies for parastomal varices

Parastomal varices develop as a consequence of portosystemic collateral formation around the stoma in the setting of portal hypertension. In the present case, angiography demonstrated tortuous, dilated distal branches of the inferior mesenteric vein surrounding the colostomy. Postoperative adhesions, scar formation, and altered anatomy may have further promoted the concentration of collateral vessels at the stomal site (18, 19).

Direct portal venous pressure measured 35 cm H_2_O (approximately 25.7 mmHg), confirming clinically significant portal hypertension. Although the HVPG measurement remains the reference standard for the hemodynamic assessment of portal hypertension, direct portal venous pressure measurement also provides objective evidence of markedly elevated portal pressure (20).

No universally accepted treatment algorithm exists for parastomal variceal hemorrhage. Local therapeutic measures, including manual compression, suturing, and sclerotherapy, may achieve temporary hemostasis but do not address the underlying portal hypertension. Non-selective beta-blockers can reduce portal pressure; however, pharmacological therapy alone is frequently insufficient to prevent recurrent ectopic variceal hemorrhage (2, 21).

Surgical revision or relocation of the stoma has been associated with substantial recurrence rates because the underlying portal hypertension remains uncorrected (2). Coil embolization and local sclerotherapy directly obliterate bleeding collateral vessels but may be followed by recurrent hemorrhage owing to recanalization or the development of new collateral pathways (21, 22). In patients with recurrent or severe variceal bleeding, TIPS placement provides sustained portal decompression and remains an important therapeutic option. Patient selection should consider hepatic functional reserve, procedural risk, the risk of hepatic encephalopathy, bleeding severity, and patient preference (2, 18, 23).

In selected patients with a localized bleeding source and technically accessible collateral vessels, transvenous embolization or local sclerotherapy may provide a less invasive therapeutic alternative (18).

In the present case, local intervention was considered an appropriate initial management strategy because the bleeding source was anatomically localized, hepatic synthetic function was preserved, the initial bleeding episodes were not immediately life-threatening, and the patient expressed reservations regarding more invasive procedures. Inferior mesenteric vein embolization and foam sclerotherapy were performed in combination with carvedilol therapy and treatment for AIH. After gastroesophageal variceal hemorrhage occurred in January 2025, TIPS was reconsidered; however, the patient declined the procedure because of concerns regarding procedural risk and uncertain clinical benefit.

Following embolization and sclerotherapy, the frequency of parastomal variceal hemorrhage decreased from one to two episodes daily to approximately one episode every 2 months, with an approximately 18-month hemorrhage-free interval. Although this outcome represents clinically meaningful improvement, it also indicates that local obliteration of collateral vessels did not eliminate the underlying portal hypertension.

Management of AIH was further limited by incomplete follow-up data and the patient's treatment preference. Prednisone was initiated at 40 mg/day, tapered by 5 mg weekly after the initial 2 weeks, and maintained at 12.5 mg/day. TPMT testing was not performed, and neither azathioprine nor mycophenolate mofetil was initiated because the patient preferred corticosteroid monotherapy after discussion of the potential adverse effects. Although serum bilirubin, albumin, and the INR remained generally preserved, intermittent elevations in aminotransferase levels persisted. Because serial IgG measurements and a complete contemporaneous biochemical dataset were unavailable in the medical records reviewed for this report, complete biochemical remission and the adequacy of long-term immunosuppressive therapy could not be confirmed.

### 3.3 Clinical implications and future directions

Oxaliplatin-associated sinusoidal injury and non-cirrhotic portal hypertension may develop insidiously. Monitoring liver biochemical parameters, platelet count, spleen size, and radiological evidence of collateral vessel formation during and after prolonged or cumulative oxaliplatin exposure may facilitate early identification of patients who warrant further hepatological evaluation (4, 5, 10).

Observational and translational studies have indicated that bevacizumab may attenuate oxaliplatin-associated sinusoidal liver injury, potentially through modulation of vascular endothelial growth factor-mediated pathways (24–26). However, the available evidence does not support the use of bevacizumab for the treatment of established portal hypertension or portosinusoidal vascular disease.

When autoimmune features are present, the diagnostic evaluation should include quantitative serum IgG measurement, appropriately interpreted autoantibody testing, exclusion of viral hepatitis, and expert histopathological review. Autoimmune investigations performed near the onset of liver injury are particularly important because results obtained years later may not reliably define the mechanism underlying the initial hepatic injury. Serial measurements of serum IgG concentrations and liver biochemical parameters are also essential for assessing the biochemical response to immunosuppressive therapy.

This case highlights the importance of maintaining a high index of suspicion for liver injury and subsequent portal hypertensive complications in patients receiving oxaliplatin, particularly when unexplained aminotransferase elevations, progressive splenomegaly, thrombocytopenia, or collateral vessel formation are observed. Determination of the underlying etiology becomes more challenging when autoimmune liver disease coexists. Management should integrate control of the bleeding source, treatment of the underlying liver disease, sustained reduction of portal pressure, and timely reassessment of TIPS placement when bleeding recurs or becomes severe.

### 3.4 Patient perspective

The patient reported that repeated hospitalizations for liver injury and recurrent parastomal bleeding had a substantial psychological impact and adversely affected quality of life. Although embolization and sclerotherapy reduced the frequency of bleeding episodes, the possibility of recurrence remained a source of ongoing concern. Following discussion with the treating team, the patient expressed a preference for continued medical management because of concerns regarding invasive diagnostic and therapeutic procedures.

## 4 Conclusion

This case describes biopsy-supported AIH developing following oxaliplatin-containing chemotherapy, followed by clinically significant multifactorial non-cirrhotic portal hypertension complicated by recurrent parastomal variceal hemorrhage and subsequent gastroesophageal variceal hemorrhage. Because the available liver biopsy did not demonstrate diagnostic histopathological features of PSVD, and comprehensive fibrosis assessment was unavailable, the findings do not support a diagnosis of histologically confirmed oxaliplatin-induced PSVD. Instead, the overall clinical presentation is most consistent with multifactorial non-cirrhotic portal hypertension associated with AIH and prior oxaliplatin exposure, with a possible contribution from oxaliplatin-associated sinusoidal or vascular liver injury. Local embolization and sclerotherapy substantially reduced the frequency of parastomal variceal hemorrhage but did not resolve the underlying portal hypertension. This case highlights the importance of individualized multidisciplinary management and careful interpretation of diagnostic uncertainty when autoimmune liver disease, chemotherapy-associated hepatic injury, and non-cirrhotic portal hypertension coexist.

## Data Availability

The raw data supporting the conclusions of this article will be made available by the authors, without undue reservation.

## Supplementary material

Supplementary Figure S1 Histopathological findings of the liver biopsy.

Supplementary Figure S2 Portal venography demonstrating dilated and tortuous distal branches of the inferior mesenteric vein surrounding the colostomy. The white arrow indicates clustered parastomal varices.

Supplementary Figure S3 Longitudinal changes in red blood cell count, platelet count, and hemoglobin level.
