# Supplementary material for: Case Report: Autoimmune hepatitis following oxaliplatin exposure presenting with multifactorial non-cirrhotic portal hypertension and recurrent parastomal variceal bleeding
Source: Front Med (Lausanne). 2026 Sep 4;13:1857471. doi: 10.3389/fmed.2026.1857471 (PMC13585593; doi:10.3389/fmed.2026.1857471)
Supplement: Supplementary file 1 [file Datasheet1.docx]

**Supplementary Table 1. Laboratory test results of the patient**

| **Item** | **20160602** | **20161129** | **20161202** | **20171225** | **20190627** | **20190707** | **20190709** | **20190712** | **20190805** | **20190821** | **20190827** | **20190916** |
| --- | --- | --- | --- | --- | --- | --- | --- | --- | --- | --- | --- | --- |
| TBI(ummol/L) | 6.7 | 8.8 |  | 5.6 | 14.3 | 15.2 | 13.5 | 14.8 | 10.20 | 17.20 | 5.90 | 44.50 |
| DBI(ummol/L) | 1.9 | 2 |  | 1.6 | 5.8 | 6.9 | 6.7 | 7.4 | 3.00 | 4.60 | 2.80 | 30.40 |
| IBI(ummol/L) | 4.8 | 6.8 |  | 4 | 8.5 | 8.3 | 6.8 | 7.4 | 7.20 | 12.60 | 3.10 | 14.10 |
| ALT(U/L) | 8 | 9 |  | 10 | 370 | 141 | 67 | 61 | 144.00 | 64.00 | 22.00 | 910.00 |
| AST(U/L) | 18 | 15 |  | 20 | 239 | 92 | 43 | 54 | 118.00 | 33.00 | 24.00 | 1151.00 |
| TP(g/L） | 71.7 | 71.9 |  | 74.4 | 83.5 | 55.2 | 49.6 | 61.6 | 86.30 | 70.70 | 59.20 | 90.30 |
| ALB(g/L） | 39.7 | 36.9 |  | 46.5 | 45.5 | 26.8 | 28.4 | 39.2 | 43.10 | 42.20 | 36.70 | 39.20 |
| GLB(g/L） | 32 | 35 |  | 27.9 | 38 | 28.4 | 21.2 | 22.4 | 43.20 | 28.50 | 22.50 | 51.10 |
| AST/ALT | 2.25 | 1.67 |  | 2 | 0.65 | 0.65 | 0.64 | 0.89 | 0.82 | 0.52 | 1.09 | 1.26 |
| ALP(U/L) |  |  |  |  | 131 |  | 58 | 88 |  |  | 54.00 |  |
| GGT(U/L) |  |  |  |  | 127 |  |  | 88 |  |  | 44.00 |  |
| PLT(10^9/L) | 276 | 261 | 255 | 315 | 362 | 269 | 186 | 282 | 386.00 | 406.00 | 253.00 | 281.00 |
| INR | 0.94 | 0.93 |  |  | 1.06 |  |  |  | 1.08 | 1.22 |  | 0.97 |

| **Item** | **20191009** | **20191013** | **20191017** | **20191104** | **20191107** | **20191128** | **20191203** | **20191205** | **20191209** | **20200303** | **20200306** | **20200602** |
| --- | --- | --- | --- | --- | --- | --- | --- | --- | --- | --- | --- | --- |
| TBI(ummol/L) | 14.70 | 10.90 | 9.10 | 13.00 |  | 10.40 | 12.00 | 5.30 | 8.00 | 12.60 |  | 16.00 |
| DBI(ummol/L) | 9.70 | 6.80 | 5.70 | 6.20 |  | 4.60 | 4.50 | 3.20 | 3.90 | 5.30 |  | 8.40 |
| IBI(ummol/L) | 5.00 | 4.10 | 3.40 | 6.80 |  | 5.80 | 7.50 | 2.10 | 4.00 | 7.30 |  | 7.60 |
| ALT(U/L) | 716.00 | 365.00 | 189.00 | 637.00 |  | 839.00 | 330.00 | 178.00 | 116.00 | 279.00 |  | 144.00 |
| AST(U/L) | 706.00 | 207.00 | 121.00 | 528.00 |  | 703.00 | 154.00 | 86.00 | 77.00 | 236.00 |  | 445.00 |
| TP(g/L） | 90.90 | 82.50 | 89.20 | 91.00 |  | 91.00 | 93.50 | 77.20 | 92.50 | 103.70 |  | 90.30 |
| ALB(g/L） | 40.90 | 37.80 | 37.70 | 44.20 |  | 39.20 | 39.20 | 31.00 | 39.90 | 43.10 |  | 34.30 |
| GLB(g/L） | 50.00 | 44.70 | 51.50 | 46.80 |  | 51.80 | 54.30 | 46.20 | 52.60 | 60.60 |  | 56.00 |
| AST/ALT | 0.99 | 0.57 | 0.64 | 0.83 |  | 0.84 | 0.47 | 0.48 | 0.66 | 0.85 |  | 3.09 |
| ALP(U/L) | 256.00 | 218.00 |  | 210.00 |  | 252.00 | 222.00 |  | 187.00 | 133.00 |  | 158.00 |
| GGT(U/L) | 316.00 | 244.00 |  | 176.00 |  | 184.00 | 163.00 |  | 139.00 | 116.00 |  | 182.00 |
| PLT(10^9/L) | 361.00 | 324.00 | 327.00 | 202.00 | 161.00 | 193.00 |  |  | 213.00 | 286.00 |  | 189.00 |
| INR | 0.91 |  | 0.90 | 0.95 |  | 0.97 |  |  |  |  |  | 1.07 |
| ANA |  |  |  |  |  |  |  |  |  |  | （±） |  |
| Anti-AMA-M2(RU/mL) |  |  |  |  |  |  |  |  |  |  | (-) |  |
| Anti-SLA |  |  |  |  |  |  |  |  |  |  | （+） |  |
| Anti-Ro-52 |  |  |  |  |  |  |  |  |  |  | (+) |  |
| Anti-gp210 |  |  |  |  |  |  |  |  |  |  | (-) |  |

| **Item** | **20210119** | **20211101** | **20220322** | **20220516** | **20220524** | **20220719** | **20220830** | **20240310** | **20250309** |
| --- | --- | --- | --- | --- | --- | --- | --- | --- | --- |
| TBI(ummol/L) | 8.80 | 11.40 | 6.30 | 7.30 |  | 14.00 | 8.60 | 7.00 | 8.10 |
| DBI(ummol/L) | 3.00 | 3.50 | 2.60 | 2.20 |  | 3.90 | 3.30 | 3.00 | 3.40 |
| IBI(ummol/L) | 5.80 | 7.90 | 3.70 | 5.10 |  | 10.10 | 5.30 | 4.00 | 4.70 |
| ALT(U/L) | 66.00 | 80.00 | 148.00 | 71.00 |  | 39.00 | 184.00 | 36.00 | 82.00 |
| AST(U/L) | 63.00 | 69.00 | 124.00 | 65.00 |  | 48.00 | 131.00 | 40.00 | 87.00 |
| TP(g/L） | 89.50 | 96.40 | 96.50 | 99.30 |  | 92.70 | 95.20 | 86.90 | 84.40 |
| ALB(g/L） | 38.70 | 37.00 | 37.20 | 38.60 |  | 39.30 | 44.60 | 41.70 | 36.10 |
| GLB(g/L） | 50.80 | 59.40 | 59.30 | 60.70 |  | 53.40 | 50.60 | 45.20 | 48.30 |
| AST/ALT | 0.95 | 0.86 | 0.84 | 0.92 |  | 1.23 | 0.71 | 1.11 | 1.06 |
| ALP(U/L) | 87.00 | 94.00 | 88.00 | 92.00 |  | 84.00 | 100.00 | 96.00 | 82.00 |
| GGT(U/L) | 18.00 | 22.00 | 20.00 | 21.00 |  | 32.00 | 26.00 | 42.00 | 44.00 |
| PLT(10^9/L) | 155.00 | 198.00 |  | 145.00 | 125.00 | 144.00 | 138.00 | 143.00 | 121.00 |
| INR | 1.07 | 1.06 |  |  |  |  |  |  |  |
| ANA |  | （±） |  |  |  |  |  |  |  |
| Anti-AMA-M2(RU/mL) |  | (-)  40.42 |  |  |  |  |  |  |  |
| Anti-SLA |  | （+） |  |  |  |  |  |  |  |
| Anti-Ro-52 |  | (+) |  |  |  |  |  |  |  |
| Anti-gp210 |  | (-) |  |  |  |  |  |  |  |

Abbreviations: ALT, alanine aminotransferase; AST, aspartate aminotransferase; GGT, gamma‑glutamyl transferase; TBI, total bilirubin; DBI, direct bilirubin; IBI, indirect bilirubin; ALT, alanine aminotransferase; AST, aspartate aminotransferase; TP, total protein; ALB, albumin; GLB, globulin; ALP, alkaline phosphatase; GGT, gamma‑glutamyl transferase; PLT, platelets; INR, international normalized ratio.

**Supplementary Table 2:** **The simplified criteria for the diagnosis of AIH – update of serological criteri**

| **Variable** | **Cutoff** | **Points** |
| --- | --- | --- |
| ANA or SMA/F-Actin | Positive^1^ | 1 |
| ANA or SMA/F-Actin | Strongly positive^2^ |  |
| or LKM | ≥1:40 | 2 |
| or SLA | Positive |  |
| IgG | >Upper normal limit | 1 |
|  | >1.1 times upper normal limit | 2 |
| Liver histology (with evidence of | Compatible with AIH | 1 |
| hepatitis) | Typical AIH | 2 |
| Absence of viral hepatitis | Yes | 2 |
|  |  | ≥6: probable AIH |
|  |  | ≥7: definite AIH |

^1^IFT: ≥1:40 when assessed on tissue sections; ≥1:80 or 1:160 for ANA when assessed on HEp-2 cells, depending on local standards. ELISA with cut-offs validated locally;

^2^IFT: ≥1:80 when assessed on tissue sections; ≥1:160 or 1:320 for ANA when assessed on HEp-2 cells. ELISA with cut-offs validated locally;

Note: if ELISA-based autoantibody assessment is negative despite of a high clinical suspicion for autoimmune hepatitis, IFT should be performed.
